# The Impact of Changing the CMS Nursing Home Compare 5-Star Ratings on the Use of Antipsychotics

**DOI:** 10.1093/geroni/igaa057.141

**Published:** 2020-12-16

**Authors:** Shekinah Fashaw, Theresa Shireman, Ellen McCreedy, Jessica Ogarek

**Affiliations:** 1 Brown University, Providence, Rhode Island, United States; 2 Brown University, PROVIDENCE, Rhode Island, United States

## Abstract

Due to their potential to increase falls and death in people with dementia, antipsychotic medications (APMs) have been the subject of several federal efforts to reduce their use in nursing homes (NHs). In 2015, the Centers for Medicare & Medicaid Services added inappropriate APM use to their NH 5-star quality ratings. We examined the impact of this policy decision on NH residents with dementia by race/ethnicity. Using a quasi-experimental study design and Minimum Data Set (MDS) 3.0 assessments, we examined long-stay NH residents with dementia. We examined changes in APM use quarterly (2013-2016) using interrupted time series analyses, stratified by race/ethnicity. There were about 1 million NH residents per quarter. Baseline use of APMs among persons with dementia was 29.1% for Whites, 29.2% for Blacks, and 33.7% for Hispanics. All three races experienced significant declines in APM use prior to the addition of AP use into the quality rating (p<0.001). During the first quarter of rating system changes, there were significant declines in APM use for all three races: Blacks, 0.48%; Hispanics, 1.0%; Whites, 0.49%. Subsequent rates of decrease in APM use did not differ from the baseline rate of decline (p> 0.5). The policy change did result in a one-time, significant drop in APM use, but did not alter the rate of decline already in place, presumably stemming from the National Partnership instituted in 2012. Hispanics started with the highest rate of APM use and experienced the greatest decreases over time and with the new star rating measure.

